# GRPR Drives Metastasis via CRABP2 and FNDC4 Pathways in Lung Adenocarcinoma

**DOI:** 10.3390/cells13242128

**Published:** 2024-12-23

**Authors:** Dong-Gun Kim, Eun-Young Choi, Hye-Mi Ahn, Youn-Jae Kim

**Affiliations:** Targeted Therapy Branch, Division of Rare and Refractory Cancer, Research Institute, National Cancer Center, Goyang 10408, Republic of Korea; kite79@ncc.re.kr (D.-G.K.); eyc@ncc.re.kr (E.-Y.C.); cocohm2801@gmail.com (H.-M.A.)

**Keywords:** lung adenocarcinoma, metastasis, GRPR, AKT, CRAPB2, FNCD4

## Abstract

Metastasis is a leading cause of lung adenocarcinoma (LUAD)-related mortality and presents significant challenges for treatment. The gastrin-releasing peptide receptor (GRPR), a member of the G protein-coupled receptor (GPCR) family, has an unclear role in LUAD progression. This study aimed to investigate the function and underlying mechanisms of GRPR in LUAD metastasis. Our findings revealed that GRPR levels were significantly elevated in tumor tissues, and higher *GRPR* expression was associated with worse overall survival outcomes. Functional assays demonstrated that *GRPR* overexpression enhanced LUAD cell invasion, while *GRPR* knockdown inhibited invasion both in vitro and in vivo. RNA sequencing and gene set enrichment analysis (GSEA) identified an enrichment of metastasis-promoting genes in *GRPR*-overexpressing cells, with CRABP2 and FNDC4 emerging as key targets. Clinical analyses further confirmed a positive correlation between *GRPR* expression and the levels of *CRABP2* and *FNDC4* in LUAD patients. These results suggest that GRPR could serve as both a prognostic marker and a therapeutic target to inhibit metastasis in LUAD.

## 1. Introduction

Lung adenocarcinoma (LUAD) constitutes approximately 80% of non-small cell lung cancer (NSCLC) cases, which account for over 85% of all lung cancers [1]. Currently, LUAD is the leading cause of cancer-related deaths globally, with metastasis detected in nearly 50% of cases at the time of diagnosis, contributing to the high mortality rate [2,3,4]. Therefore, understanding the molecular mechanisms that drive metastasis and identifying specific biomarkers for diagnosis, prognosis, and treatment of LUAD is crucial.

G protein-coupled receptors (GPCRs) are a large family of proteins characterized by seven transmembrane domains, involved in signal detection, activation, transduction, and cellular responses [5]. They have been implicated in the pathological mechanisms of various diseases [6,7,8]. Elevated expression of *GPCR* genes has been observed in several tumor types, where they contribute to cancer progression and drug resistance [9]. The gastrin-releasing peptide receptor (GRPR), also known as bombesin receptor 2 (BB2), is a member of the GPCR family, recognized for its binding ligand, gastrin-releasing peptide (GRP) [10]. GRPR is known to regulate gastrointestinal motility, gastric emptying, and smooth muscle contraction. It is also distributed widely in nervous tissue, where it plays roles in modulating memory, cognition, circadian rhythms, and emotional behaviors [11,12]. Previous research has shown that GRPR is overexpressed in several cancers, including small cell lung cancer, breast cancer, prostate cancer, and pancreatic adenocarcinoma [13,14,15]. GRPR acts as a growth factor in an autocrine manner, playing a critical role in promoting cancer metastasis and angiogenesis in various cancers [16,17,18,19]. However, its functional role in LUAD metastasis remains poorly understood.

In this study, we identify GRPR as a key promoter of metastasis in LUAD. We evaluated GRPR expression and its prognostic significance in patients with LUAD, as well as its association with invasion and metastasis. Furthermore, we investigated the downstream mechanisms underlying the oncogenic role of GRPR in LUAD metastasis. Our findings suggest that targeting GRPR may improve therapeutic outcomes for patients with LUAD with metastatic disease.

## 2. Materials and Methods

### 2.1. Public Microarray Data Analysis

Public microarray datasets (GSE50081, GSE31210, GSE19188, GSE19804, and GSE14814) were obtained from the NCBI GEO database (http://www.ncbi.nlm.nih.gov/geo/) (accessed on 18 December 2024). Comparative and survival analyses were conducted using LUNG CANCER EXPLORER (https://lce.biohpc.swmed.edu/lungcancer/index.php) (accessed on 18 December 2024) and the Kaplan–Meier Plotter (https://kmplot.com/analysis/) (accessed on 18 December 2024) [20,21]. Correlation analysis was performed using R (http://www.r-project.org) (accessed on 18 December 2024), and graphs and heatmaps were generated using Microsoft Excel 2010 and R 4.4.1.

### 2.2. Antibodies and Reagents

The primary antibody mouse anti-FLAG-M2 (F1804) was purchased from Sigma-Aldrich (St. Louis, MO, USA). Rabbit anti-GRPR (PA5-26791) was purchased from Invitrogen (Waltham, MA, USA). Rabbit anti-CRABP2 (10225-1-AP) was sourced from Proteintech (Rosemont, IL, USA), and rabbit anti-FNDC4 (GTX46001) from GeneTex (Irvine, CA, USA). Rabbit anti-phospho-AKT (#9271), anti-AKT (#9272), and mouse anti-β-actin (#3700) were purchased from Cell Signaling Technology (Boston, MA, USA). RC3095 (R9653) was purchased from Sigma-Aldrich, PD176252 (#2602) from Tocris Bioscience (Bristol, UK), and LY294002 (#9901) from Cell Signaling Technology.

### 2.3. LUAD Cell Culture and Transfection

A549 Red-Firefly Luciferase (FLuc) cells were obtained from Perkin Elmer (Waltham, MA, USA), and A427 cells from the Korea Cell Line Bank (KCLB, Seoul, Republic of Korea). Both cell lines were cultured in RPMI-1640 medium (Cytiva, Marlborough, MA, USA) supplemented with 10% fetal bovine serum (Cytiva, USA) and 1% penicillin-streptomycin (Welgene, Gyeongsan, Republic of Korea) at 37 °C in a humidified atmosphere with 5% CO_2_.

For RNAi transfection, 1.5 × 10^5^ A549 or A427 cells were plated in six-well plates and incubated overnight. The cells were then transfected with targeting sequences and non-targeting controls using Lipofectamine 2000 in Opti-MEM. After 4 h of incubation, the medium was replaced with complete medium, and the cells were incubated for 48 h. After 2 days of siRNA transfection, knockdown efficiency was verified by Western blotting, and proliferation and invasion assays were performed. Sequences of the transfected siRNAs are listed in Table S1.

### 2.4. Reverse Transcription and Quantitative Real-Time PCR (qRT-PCR)

Total RNA was extracted using the RNeasy Mini Kit (QIAGEN, Hilden, Germany) according to the manufacturer’s instructions. Reverse transcription was performed using 1 μg of total RNA as a template with SuperScript™ III Reverse Transcriptase (Invitrogen, MA, USA). qRT-PCR was performed in triplicate on a LightCycler 480 (Roche, Basel, Switzerland) using SYBR Green I Master Mix (Roche, Switzerland). Gene expression levels were normalized to β-actin (ACTB), and the values are presented as the mean ± standard error of the mean (SEM) from independent experiments. Primer sequences are provided in Table S1.

### 2.5. RNA-Seq Analysis

RNA-seq libraries from the A427 cell line were prepared using 2 μg of total RNA and the TruSeq Stranded mRNA Sample Preparation Kit (Illumina, CA, USA), following the manufacturer’s protocol. Sequencing was performed on the HiSeq 2500 Sequencing System (Illumina, CA, USA), generating paired-end reads of 101 bp length. The RNA-seq read data were mapped using HISAT2, which performed short-read gapped alignment to the GRCh38 reference genome and were analyzed for count values aligned to the transcript reference using StringTie programs [22,23]. We then conducted differential gene expression analysis using DESeq2 based on these count values and performed gene set enrichment analysis (GSEA) [24].

### 2.6. Immunoblotting Analysis

Whole cell lysates were prepared using RIPA buffer (iNtRON Biotechnology, Seongnam, Republic of Korea) supplemented with a protease inhibitor cocktail (Roche, Basel, Switzerland). Protein concentrations were quantified using the Pierce BCA Protein Assay Kit (Thermo Fisher Scientific, Waltham, MA, USA). Equal amounts of protein lysates were resolved on 8–16% Bis-Tris protein gels (Invitrogen, MA, USA) and transferred onto PVDF membranes (Millipore, Burlington, MA, USA). The membranes were blocked with 5% skim milk (BD Biosciences, Milpitas, CA, USA). HRP-conjugated anti-mouse and anti-rabbit IgG antibodies (Bio-Rad, Hercules, CA, USA) were used as secondary antibodies. Protein bands were detected using the WEST-ZOL Plus Western Blot Detection System (iNtRON Biotechnology, Seongnam, Republic of Korea). Band intensities were normalized to β-actin and quantified using ImageJ (version 1.54j).

### 2.7. Invasion Assay

Transwell chambers (Corning, New York, NY, USA) were coated with Matrigel Basement Membrane Matrix (BD Biosciences, CA, USA). Cells were suspended in serum-free medium and seeded into the upper chamber at a density of 2.0–5.0 × 10^4^ cells per well, while serum-containing medium was added to the lower chamber. After 24 h of incubation, cells that had migrated through the pores were stained using the Diff-Quick staining solution (Sysmex, Kobe, Japan) and observed under a microscope.

### 2.8. Proliferation Assay

Cells were seeded in 96-well plates at a density of 2 × 10^3^ cells per well. After incubation for 0 to 72 h, a CyQUANT NF Cell Proliferation dye reagent mixture (Invitrogen, USA) was added and incubated at 37 °C for 30 min. Fluorescence intensity was measured at 530 nm (excitation) and 485 nm (emission) using an M200 Pro microplate reader (TECAN, Männedorf, Switzerland).

### 2.9. In Vivo Tumor Model

This tumor model was reviewed and approved by the Institutional Animal Care and Use Committee (IACUC) of the National Cancer Center Research Institute (NCCRI), an AAALAC International-accredited facility that follows Institute of Laboratory Resources (ILAR) guidelines. Ten five-week-old female nude mice (BALB/c-nude) were obtained from Orient Bio (Seongnam, Republic of Korea). After one week of acclimation, mice were randomly divided into two groups (WT vs. knockdown), and 1.0 × 10^6^ A549-FLuc-WT and *GRPR*-knockdown cells suspended in 100 μL of phosphate-buffered saline (PBS) were injected intravenously via the tail vein. Tumor metastasis was monitored weekly through bioluminescence imaging using the IVIS Lumina III system (PerkinElmer, Hopkinton, MA, USA). Mice were euthanized 27 days post-injection, and lung metastasis was analyzed under a microscope. The number of metastatic nodules in the lungs was counted, and lung tissues were stained with H&E to assess tumor metastasis.

### 2.10. Statistical Analysis

Statistical analyses were performed using Student’s *t*-test and log-rank test, with statistical significance set at *p* < 0.05.

## 3. Results

### 3.1. GRPR Expression Is Associated with the LUAD Patient Outcomes

**Figure 1:**
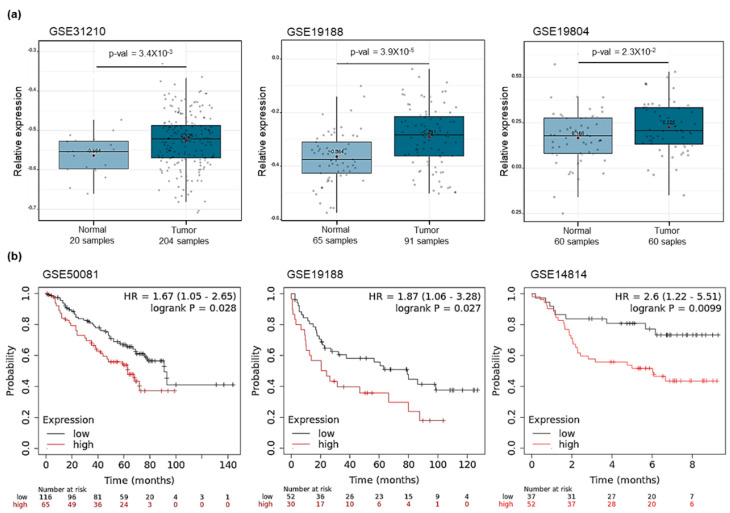
GRPR clinical analysis in LUAD. (**a**) Comparison of *GRPR* expression in normal and tumor tissues of patients of LUAD (*p* = 3.4 × 10^−3^ for GSE31210; *p* = 3.9 × 10^−5^ for GSE19188; *p* = 2.3 × 10^−2^ for GSE19804, *t*-test). (**b**) Overall survival analysis of patients with LUAD stratified by *GRPR* expression levels (*p* = 2.8 × 10^−2^ for GSE50081; *p* = 2.7 × 10^−2^ for GSE19188; *p* = 9.9 × 10^−3^ for GSE14814, log–rank test).

To assess the significance of *GRPR* gene expression in LUAD, we compared its expression levels between normal and tumor tissues. Analysis of LUAD datasets from the NCBI GEO database revealed that *GRPR* expression was significantly higher in tumor tissues than in normal tissues (*p* = 3.4 × 10^−3^ for GSE31210; *p* = 3.9 × 10^−5^ for GSE19188; *p* = 2.3 × 10^−2^ for GSE19804, *t*-test, Figure 1a). Additionally, we analyzed survival data to investigate the correlation between *GRPR* expression and patient outcomes (Figure 1b). In datasets GSE50081, GSE19188, and GSE14814, higher *GRPR* expression was associated with poorer overall survival rates (*p* = 2.8 × 10^−2^ for GSE50081 (*N* = 181); *p* = 2.7 × 10^−2^ for GSE19188 (n = 82); *p* = 9.9 × 10^−3^ for GSE14814 (n = 89), log-rank test). These results suggest that *GRPR* overexpression may promote LUAD tumorigenesis and progression.

### 3.2. GRPR Expression Regulates LUAD Cell Invasion

**Figure 2:**
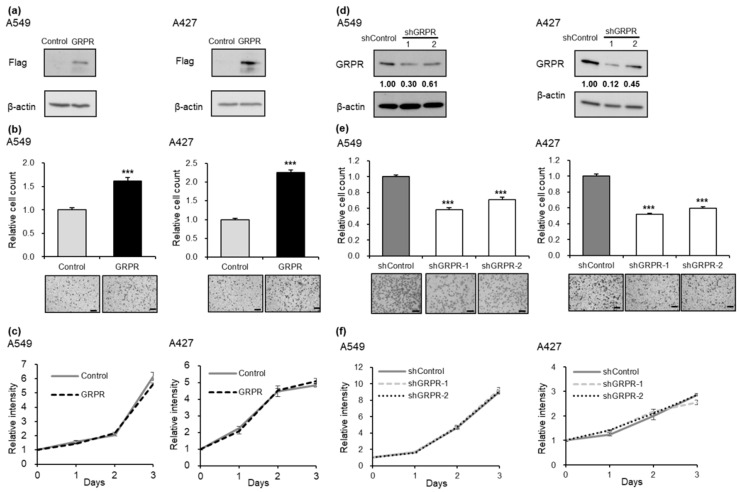
*GRPR* expression regulates the invasive properties of LUAD cells. (**a**) Flag-GRPR expression in A549 and A427 cells. (**b**,**c**) Invasion (**b**) and proliferation assay (**c**) results following *GRPR* overexpression in A549 and A427 cells. (**d**) GRPR expression in *GRPR* knockdown A549 and A427 cells. (**e**,**f**) Invasion (**e**) and proliferation assay (**f**) results following *GRPR* knockdown in A549 and A427 cells. Error bars represent the mean ± standard error of the mean (SEM); *** *p* < 0.001 vs. control and siControl, *t*-test. Scale bar = 200 μm.

To explore the role of GRPR in LUAD cells, we examined its effects on cell proliferation and invasion in Flag-tagged *GRPR*-overexpressing LUAD cell lines (Figure 2a). *GRPR* overexpression significantly enhanced cancer cell invasion (Figure 2b), although it had no impact on cell proliferation (Figure 2c). We further performed invasion and proliferation assays in LUAD cell lines with *GRPR* knockdown (Figure 2d). *GRPR* knockdown significantly inhibited lung cancer cell invasion (Figure 2e), while no changes were observed in cell proliferation following GRPR depletion (Figure 2f).

**Figure 3:**
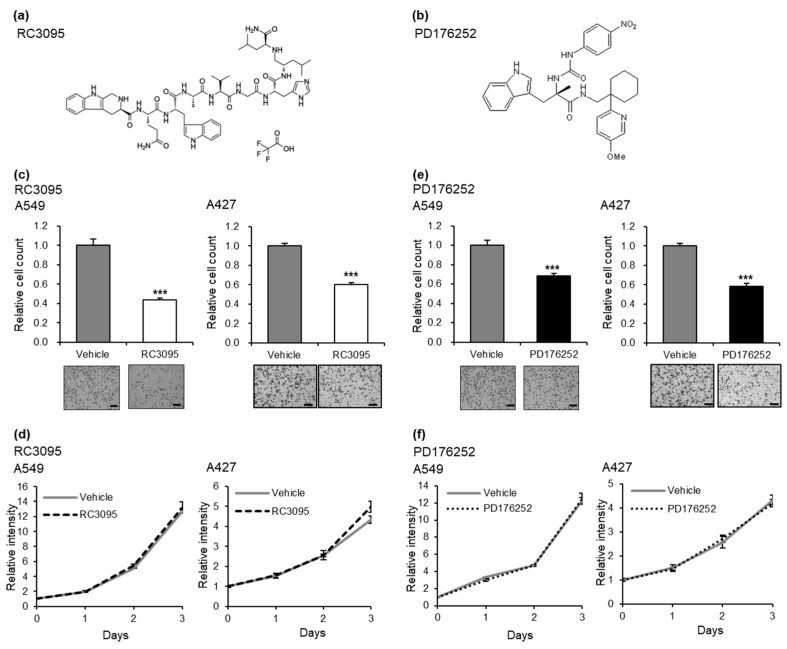
*GRPR* inhibition attenuates the invasive properties of LUAD cells. (**a**,**b**) Structures of *GRPR* inhibitors: RC3095 (**a**) and PD176252 (**b**). (**c**,**d**) Invasion (**c**) and proliferation assay (**d**) results following treatment with *GRPR* inhibitors RC3095 (1 μM) in A549 and A427 wild-type cells. (**e**,**f**) Invasion (**e**) and proliferation assay (**f**) results following treatment with *GRPR* inhibitors PD176252 (1 μM) in A549 and A427 wild-type cells. Error bars represent the mean ± standard error of the mean (SEM); *** *p* < 0.001 vs. vehicle, *t*-test. Scale bar = 200 μm.

We also evaluated the effects of GRPR inhibition on cancer cell invasion and proliferation. RC-3095, a synthetic peptide shown to inhibit GRPR activation [25], and PD176252, a potent non-peptide GRPR antagonist [26], were used in these experiments (Figure 3a,b). Treatment with RC-3095 and PD176252 markedly reduced A549 and A427 wild-type cell invasion (Figure 3c,e) but did not affect cell proliferation (Figure 3d,f). These findings indicate that *GRPR* expression plays a critical role in promoting LUAD cell invasiveness, independent of cell proliferation.

### 3.3. GRPR Overexpression Induces Metastasis-Related Gene Expression in LUAD Cells

**Figure 4:**
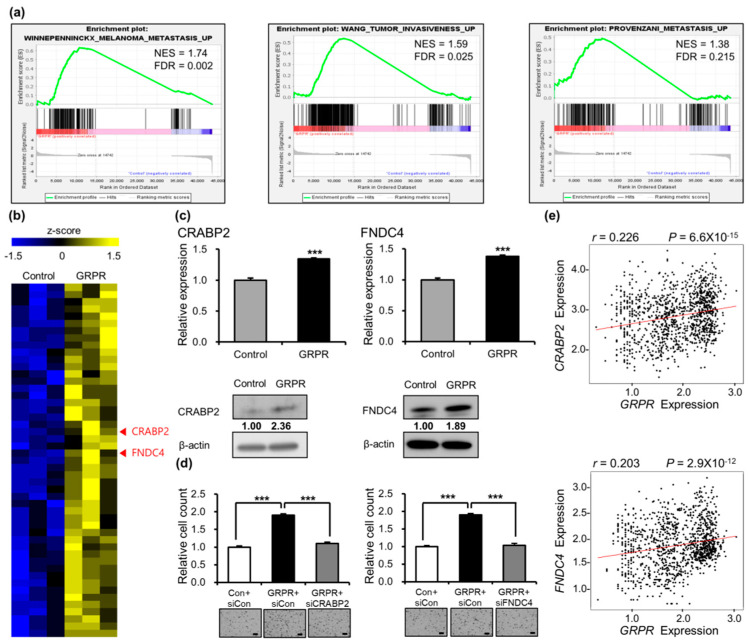
*CRABP2* and *FNDC4* are target genes of *GRPR* signaling. (**a**) Gene set enrichment analysis (GSEA) of RNA-seq data after *GRPR* overexpression in A427 cells. (**b**) Heatmap displaying changes in the expression of putative target genes. (**c**) *CRABP2* and *FNDC4* gene and protein expression levels following *GRPR* overexpression in A427 cells. (**d**) Invasion assay results after knockdown of *CRABP2* or *FNDC4* in the context of *GRPR* overexpression in A427 cells. (**e**) Correlation between *GRPR* and *CRABP2* or *FNDC4* expression in patients with LUAD (*p* = 6.6 × 10^−15^ for *CRABP2*; *p* = 2.9 × 10^−12^ for *FNDC4*, *t*-test). Error bars represent the mean ± standard error of the mean (SEM); *** *p* < 0.001 vs. control, *t*-test. Scale bar = 200 μm.

To investigate the relationship between *GRPR* overexpression and metastasis-related genes, we performed RNA sequencing on A427 cells overexpressing *GRPR* and conducted gene set enrichment analysis (GSEA). We performed GSEA by selecting a set of 23 genes that had been used in previous metastasis studies and were deposited in MSigDB (Supplementary Table S2). Notably, genes promoting metastasis in various cancer types were significantly enriched in the *GRPR*-overexpressing cells (FDR < 0.25, Figure 4a).

We further analyzed differentially expressed genes (DEGs) and identified 49 coding genes that were significantly upregulated in *GRPR*-overexpressing cells. From these, we selected cellular retinoic acid-binding protein 2 (CRABP2) and fibronectin type III domain containing 4 (FNDC4) as key target genes, which have been implicated in metastasis in previous studies (Figure 4b). CRABP2 is a small cytosolic protein in the lipid-binding protein family that transports retinoic acid from the cytoplasm to the nucleus [27]. FNDC4 is a secreted protein known for its role as a crucial anti-inflammatory factor [28]. Previous studies have demonstrated that overexpression of *CRABP2* and *FNDC4* can enhance cell invasion and metastasis in various cancers [29,30,31,32]. We validated *CRABP2* and *FNDC4* levels in A427 cells overexpressing *GRPR*. We found that *GRPR* overexpression led to a significant increase in the transcription and protein levels of both genes (Figure 4c), and *GRPR* knockdown weakly but significantly reduced both gene expressions (Figure S1a,b). To further examine their role in LUAD cells, we knocked down *CRABP2* or *FNDC4* expression (Figure S2a,b) and observed a significant reduction in cell invasion (Figure S2c,d). Moreover, the enhanced invasion seen in *GRPR*-overexpressing A427 cells was reversed by *CRABP2* or *FNDC4* knockdown (Figure 4d).

To assess the correlation between *GRPR* expression and *CRABP2* or *FNDC4* expression in patients with LUAD, we performed a correlational analysis using KM plotter data [21]. A weak positive correlation was observed between *GRPR* and *CRABP2* or *FNDC4* expression in patients with LUAD (*r* = 0.226, *p* = 6.6 × 10^−15^ for CRABP2; *r* = 0.203, *p* = 2.9 × 10^−12^ for FNDC4, Pearson correlation coefficient, Figure 4e). In summary, these findings suggest that the increased invasiveness of LUAD cells driven by *GRPR* overexpression is mediated by the upregulation of *CRABP2* and *FNDC4*, two genes associated with metastatic potential.

### 3.4. GRPR-AKT Signaling Promotes the Expression of Target Genes

**Figure 5:**
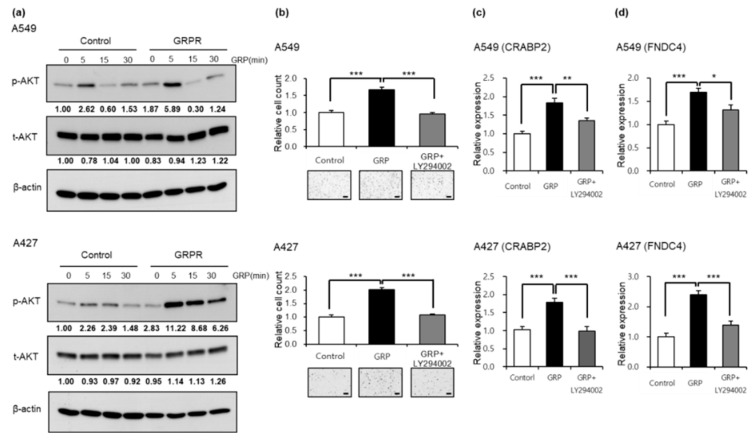
GRPR induces target gene expression via AKT signaling. (**a**) AKT phosphorylation in response to GRP treatment (100 nM), with or without *GRPR* overexpression, in A549 and A427 cells. (**b**) Invasion assay results following GRP and LY294002 (20 μM) treatments in A549 and A427 wild-type cells. (**c**,**d**) *CRABP2* (**c**) and *FNDC4* (**d**) gene expression levels after GRP and LY294002 treatment in A549 and A427 wild-type cells. Error bars represent the mean ± standard error of the mean (SEM); * *p* < 0.05, ** *p* < 0.01, *** *p* < 0.001 vs. control, *t*-test. Scale bar = 200 μm.

Previous studies have demonstrated that GRPR regulates downstream targets through activation of the PI3K/AKT pathway [33,34,35,36]. To evaluate the relationship between *GRPR* overexpression and AKT signaling in LUAD cells, we analyzed GRP-induced AKT phosphorylation in a time-dependent manner. *GRPR* overexpression resulted in increased AKT phosphorylation upon GRP treatment in the A549 and A427 cell lines compared to the control (Figure 5a). Furthermore, GRP treatment enhanced cellular invasion, which was significantly reduced to baseline levels when co-treated with LY294002, a potent PI3K/AKT inhibitor (Figure 5b). Notably, GRP treatment also upregulated expression of *CRABP2* and *FNDC4* in A549 and A427 wild-type cell lines, while inhibition of AKT signaling led to a decrease in the expression of these genes (Figure 5c,d). These results suggest that GRPR-induced upregulation of LUAD cell invasion is mediated through AKT signaling.

### 3.5. GRPR Knockdown Reduces the Metastasis of LUAD Cells

**Figure 6:**
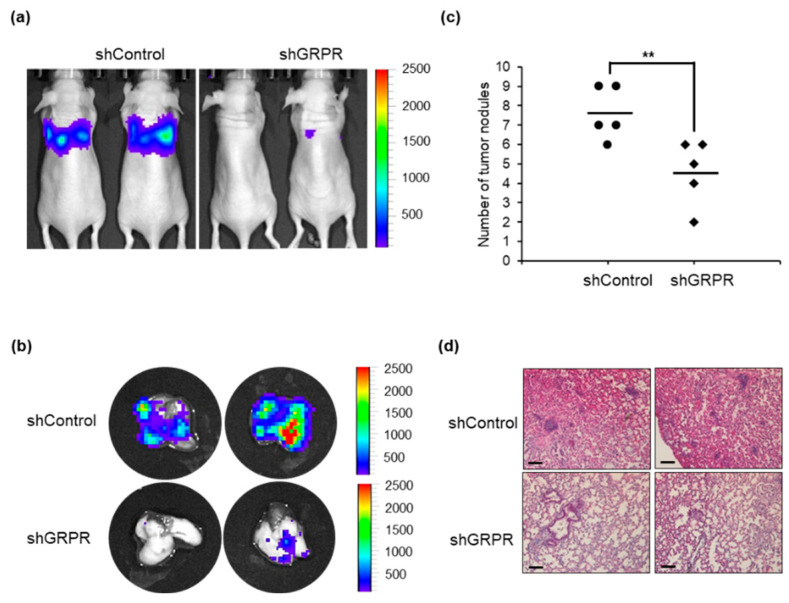
Knockdown of *GRPR* inhibits LUAD metastasis in a mouse model. (**a**) Luminescence signal of A549-FLuc cells in the lungs of mice 4 weeks after intravenous injection. (**b**) Luminescence signal of A549-FLuc cells in the lungs of mice post-sacrifice. Quantification of these results is shown in Figure S3b. (**c**) Number of metastatic nodules in the lungs of mice post-sacrifice. (**d**) Hematoxylin and eosin (H&E) staining of lung tissues from mice; ** *p* < 0.01 vs. shControl, *t*-test. Scale bar = 200 μm.

We further explored the effects of *GRPR* knockdown on the metastatic potential of LUAD cells in vivo. *GRPR*-silenced A549-FLuc cells were injected intravenously into the lateral tail veins of mice, and tumor metastasis was monitored weekly via luminescence imaging. Mice injected with *GRPR*-silenced A549-FLuc cells exhibited significantly reduced lung metastasis (Figure 6a and Figure S3a). Metastatic nodules and metastatic burden in the lungs were markedly decreased following *GRPR* knockdown in LUAD cells (Figure 6b,c and Figure S3b). Additionally, H&E staining of lung tissues (Figure 6d) revealed a reduction in metastatic tumor regions in mice with *GRPR*-silenced cells. Collectively, these findings suggest that reducing *GRPR* expression can inhibit LUAD metastasis.

## 4. Discussion

Lung cancer remains a leading cause of mortality worldwide, responsible for over one million deaths annually, with metastasis closely associated with high mortality rates [3,37]. Despite advances in surgical intervention, radiotherapy, and multimodal treatments, lung cancer incidence continues to rise, and *GRPR* overexpression is found in 68% of adenocarcinoma cases in NSCLC [38,39].

GRPR is widely expressed in various cancers, and its association with poor prognosis in cancer patients is well established. Inhibiting GRPR signaling has been shown to reduce cancer cell growth and viability [40,41]. Furthermore, GRPR activation has been linked to metastasis in prostate, colon, and breast cancers, though the specific mechanisms by which GRPR promotes metastasis are not yet fully understood [42,43]. This makes GRPR an appealing target for cancer therapy and diagnosis. Elucidating the role of GRPR in cancer metastasis is crucial for developing effective therapeutic strategies for patients with metastatic cancer. Our research demonstrated that GRPR levels in LUAD cells were significantly higher than in normal cells, and elevated *GRPR* expression negatively impacted the prognosis of LUAD patients. Moreover, we showed that *GRPR* overexpression upregulates genes involved in metastasis, highlighting a close link between *GRPR* expression and LUAD progression, suggesting that GRPR could serve as a prognostic marker in LUAD.

*GRPR* overexpression significantly enhanced the invasive capacity of LUAD cells. Conversely, *GRPR* suppression markedly reduced invasive capacity in both in vitro and in vivo models. Regarding the molecular background of LUAD, KRAS mutations are the predominant driver mutations (25%), followed by EGFR mutations (23%) [44]. In our study, we used KRAS mutant cell lines A549 and A427. The role of GRPR in cell lines with other driver mutations, including EGFR, needs to be discovered in future studies.

Notably, GRPR inhibition using antagonist drugs significantly diminished tumor invasion. RC-3095 has emerged as one of the most promising antagonists, exhibiting significant anti-proliferative effects on various cancer cells [45,46,47,48]. PD176252, the first synthesized small-molecule GRPR antagonist, has been identified as a potent receptor ligand capable of inhibiting the tumorigenesis of cancer cells [26,49]. Currently, there are no GRPR inhibitors that have received approval for clinical use. Previous studies have primarily focused on using GRPR-targeted drugs for molecular imaging and monitoring cancer recurrence or metastasis [50,51]. Some studies have explored combining GRPR antagonists with cytotoxic agents [52,53]. Our findings are the first to suggest that GRPR-targeted therapies may also serve as anti-metastatic treatments by inhibiting LUAD invasiveness.

GPCRs play a critical role in regulating transcription through the activation of signaling pathways that influence transcription factor activity. Previous research indicates that GRPR signaling induces transcription and DNA synthesis in human pancreatic adenocarcinoma cells and regulates Cox-2 mRNA expression in various tissues [54,55,56]. Consistent with these findings, our results show that GRPR induces the expression of several metastasis-related genes, including the oncogenes *CRABP2* and *FNDC4*. CRABP2 is highly expressed in docetaxel-resistant breast carcinoma and promotes invasion and metastasis in ER-negative breast cancer [57,58]. It is also overexpressed in pancreatic ductal adenocarcinoma (PDAC), where its deletion reduces cell migration and invasion by downregulating MMP-2 and MMP-14 [29]. In lung cancer, *Crabp2* expression is elevated in highly metastatic mouse lung cancer cells compared to less metastatic counterparts, facilitating integrin β1/FAK/ERK signaling [30]. Elevated FNDC4 expression is linked to poor survival in hepatocellular carcinoma, where it promotes migration and invasion via the PI3K/Akt signaling pathway [31]. Similarly, FNDC4 is associated with unfavorable outcomes in glioblastoma, enhancing cell proliferation and influencing the S phase of tumor cells [32]. Given the roles of *CRABP2* and *FNDC4* in cancer progression, they are being explored as potential biomarkers or therapeutic targets in various cancers [31,32,58,59,60]. However, no clinical trials have specifically targeted these genes in cancer treatment, and their role in LUAD remains unclear. Our study is the first to reveal that *CRABP2* and *FNDC4* are associated with *GRPR* expression in LUAD and that GRPR upregulates their expression, which enhances LUAD invasiveness. Importantly, suppression of *CRABP2* or *FNDC4* reduced cancer invasion, even in the presence of *GRPR* overexpression, suggesting that these genes play a critical role in LUAD metastasis. However, the association between the GRPR-CRABP2/FNDC4 axis and LUAD metastasis has not been validated in vivo, which is a limitation of our results that needs to be verified in future studies.

GRPR activation leads to the stimulation of PKC and regulation of the ERK, JNK, and p38 pathways, which in turn regulate the expression of genes involved in cell proliferation and differentiation [61,62]. In colon cancer, GRPR enhances invasion through the RhoA/ROCK pathway [43], while in prostate cancer, *GRPR* overexpression promotes a malignant phenotype via activation of AKT1, PKCε, TYK2, and MST1 pathways [63]. In NSCLC, GRPR activates c-Src, triggering the release of EGFR ligands and subsequently activating the PI3K/AKT pathway. GRPR also induces ROS generation, which is linked to PI3K/AKT activation and enhances cell migration in LUAD cells [34,35]. In this study, we found that *GRPR* overexpression further stimulated AKT activation through GRP, and the enhanced invasion of LUAD cells induced by GRP was inhibited by AKT signaling inhibitors. Additionally, GRP-induced upregulation of *CRABP2* and *FNDC4* was suppressed by AKT inhibition. This is the first study to demonstrate that *GRPR* overexpression and AKT phosphorylation promote LUAD invasion by upregulating *CRABP2* and *FNDC4* expression. Future studies should aim to identify the transcription factors or mechanisms through which AKT signaling enhances *CRABP2* and *FNDC4* expression.

## 5. Conclusions

This study demonstrated that *GRPR* expression is elevated in LUAD and is associated with a poor prognosis in patients. Additionally, we found that *GRPR* overexpression enhances the metastatic potential of LUAD cells by upregulating the oncogenic genes *CRABP2* and *FNDC4*. Moreover, our results indicate that the AKT signaling pathway plays a key role in regulating these target genes in response to GRPR activation. Collectively, these findings suggest that the GRPR-AKT-CRABP2/FNDC4 axis may serve as a promising therapeutic target in LUAD.

## Data Availability

All the data needed to evaluate the conclusions are presented in this paper. Additional data related to this study were requested from the authors.

## Supplementary Materials

The following supporting information can be downloaded at: https://www.mdpi.com/article/10.3390/cells13242128/s1, Figure S1: Results of RT-qPCR after knockdown of *CRABP2* and *FNDC4* in A427 cells; Figure S2: Results of Western blot, invasion, and proliferation assay after knockdown of *CRABP2* and *FNDC4* in A427 cells; Figure S3: Luminescence signal of A549-FLuc cells in the lungs of mice and quantitation of metastatic burden; Figure S4: Uncropped Western blot images of Figure 2; Figure S5: Uncropped Western blot images of Figure 4; Figure S6: Uncropped Western blot images of Figure 5; Figure S7: Uncropped Western blot images of Figure S2; Table S1: Lists of primer sequences for RT-qPCR and oligonucleotide sequence for siRNA; Table S2: Lists and results of genesets for GSEA.
