# Supplementary material for: GRPR Drives Metastasis via CRABP2 and FNDC4 Pathways in Lung Adenocarcinoma
Source: Cells. 2024 Dec 23;13(24):2128. doi: 10.3390/cells13242128 (PMC11674891; doi:10.3390/cells13242128)
Supplement: Supplementary file 1 [file cells-13-02128-s001.zip › Supplementary Figures.pptx]

## Slide 1
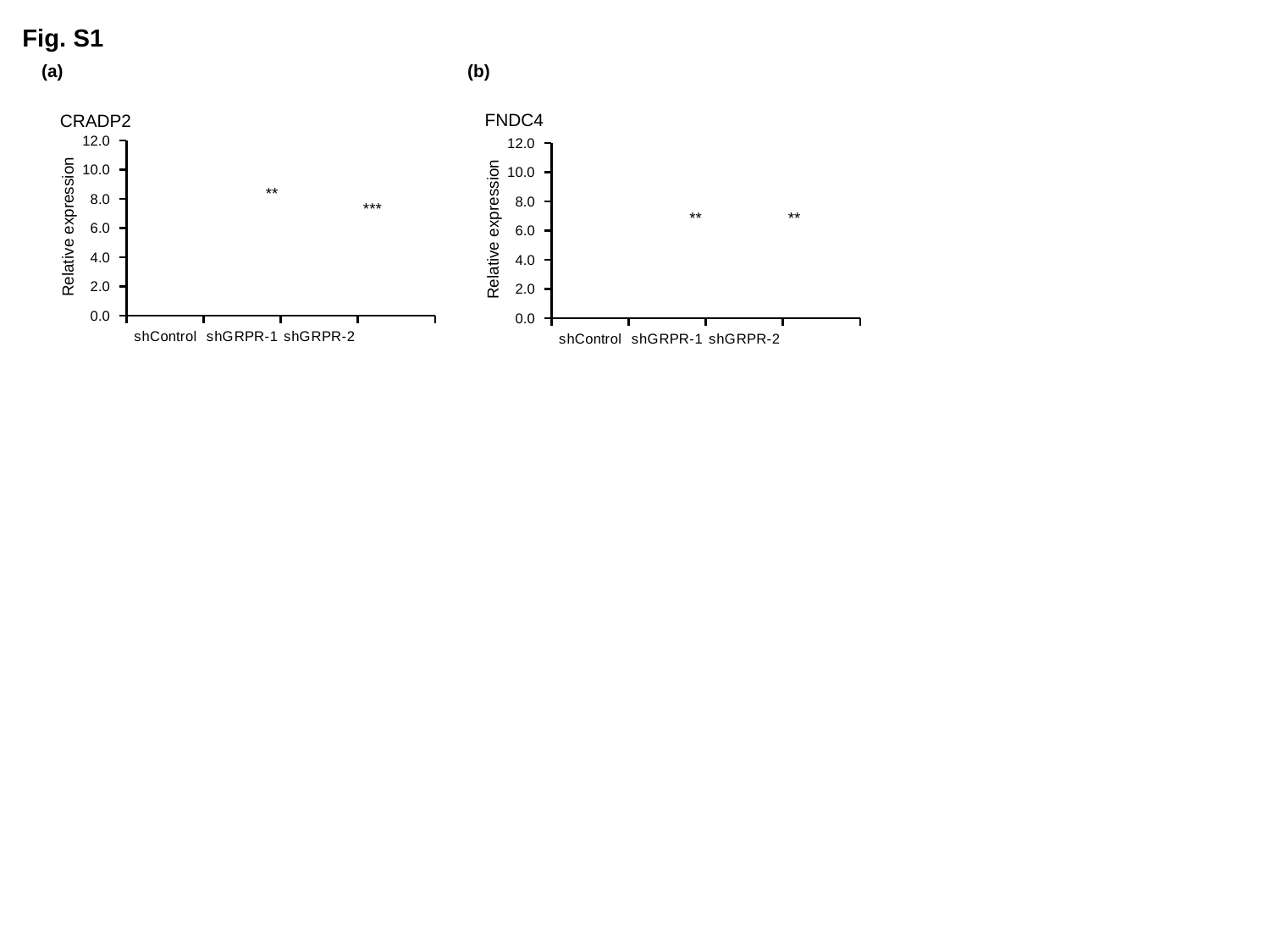

Fig. S1
(b)
(a)
FNDC4
**
**
Relative expression
### Chart
| Category | FNDC4 |
|---|---|
| shControl | 1.0031785110913038 |
| shGRPR-1 | 0.6539093537622468 |
| shGRPR-2 | 0.673641988458047 |CRADP2
**
***
Relative expression
### Chart
| Category | CRADP2 |
|---|---|
| shControl | 0.997825844897971 |
| shGRPR-1 | 0.8010827068555555 |
| shGRPR-2 | 0.6832428209815679 |

## Slide 2
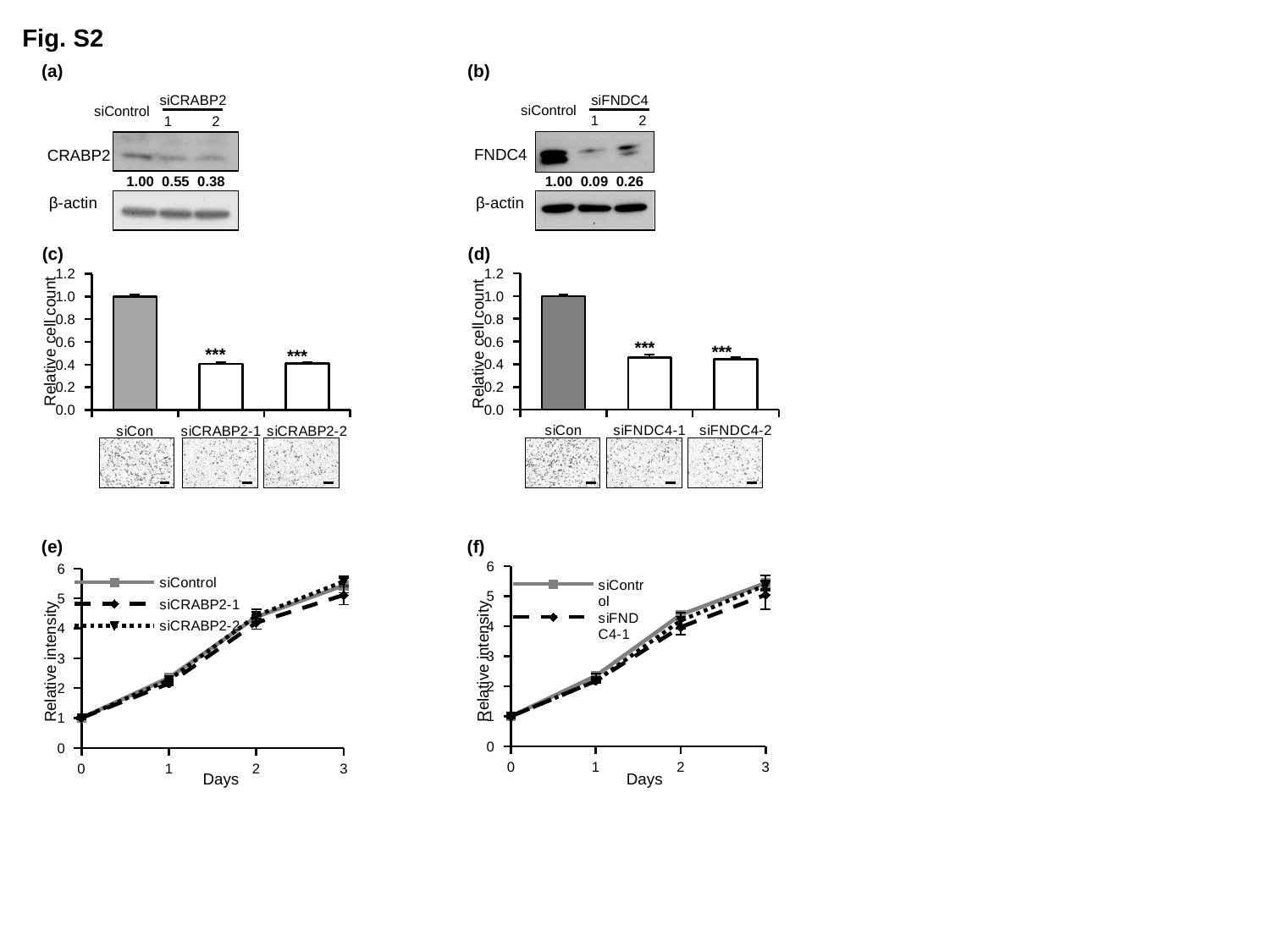

Fig. S2
(b)
(a)
siFNDC4
siCRABP2
siControl
siControl
 1 2
 1 2
FNDC4
CRABP2
1.00 0.55 0.38
1.00 0.09 0.26
β-actin
β-actin
(d)
(c)
### Chart
| Category | |
|---|---|
| siCon | 1.0 |
| siCRABP2-1 | 0.40556990040575436 |
| siCRABP2-2 | 0.4079675396532645 |Relative cell count
***
***
### Chart
| Category | |
|---|---|
| siCon | 1.0 |
| siFNDC4-1 | 0.45721136112135735 |
| siFNDC4-2 | 0.4419033566949465 |Relative cell count
***
***
(f)
(e)
### Chart
| Category | siControl | siFNDC4-1 | siFNDC4-2 |
|---|---|---|---|
| 0 | 1.0 | 1.0 | 1.0 |
| 1 | 2.344763237695358 | 2.175588235294118 | 2.1734039587398937 |
| 2 | 4.379986004198741 | 3.968235294117647 | 4.18511290772233 |
| 3 | 5.432470258922323 | 5.044117647058824 | 5.361304711458042 |Relative intensity
Days
### Chart
| Category | siControl | siCRABP2-1 | siCRABP2-2 |
|---|---|---|---|
| 0 | 1.0 | 1.0 | 1.0 |
| 1 | 2.344763237695358 | 2.1507868383404865 | 2.2704026115342764 |
| 2 | 4.379986004198741 | 4.184549356223176 | 4.424374319912949 |
| 3 | 5.432470258922323 | 5.113304721030043 | 5.563656147986943 |Relative intensity
Days

## Slide 3
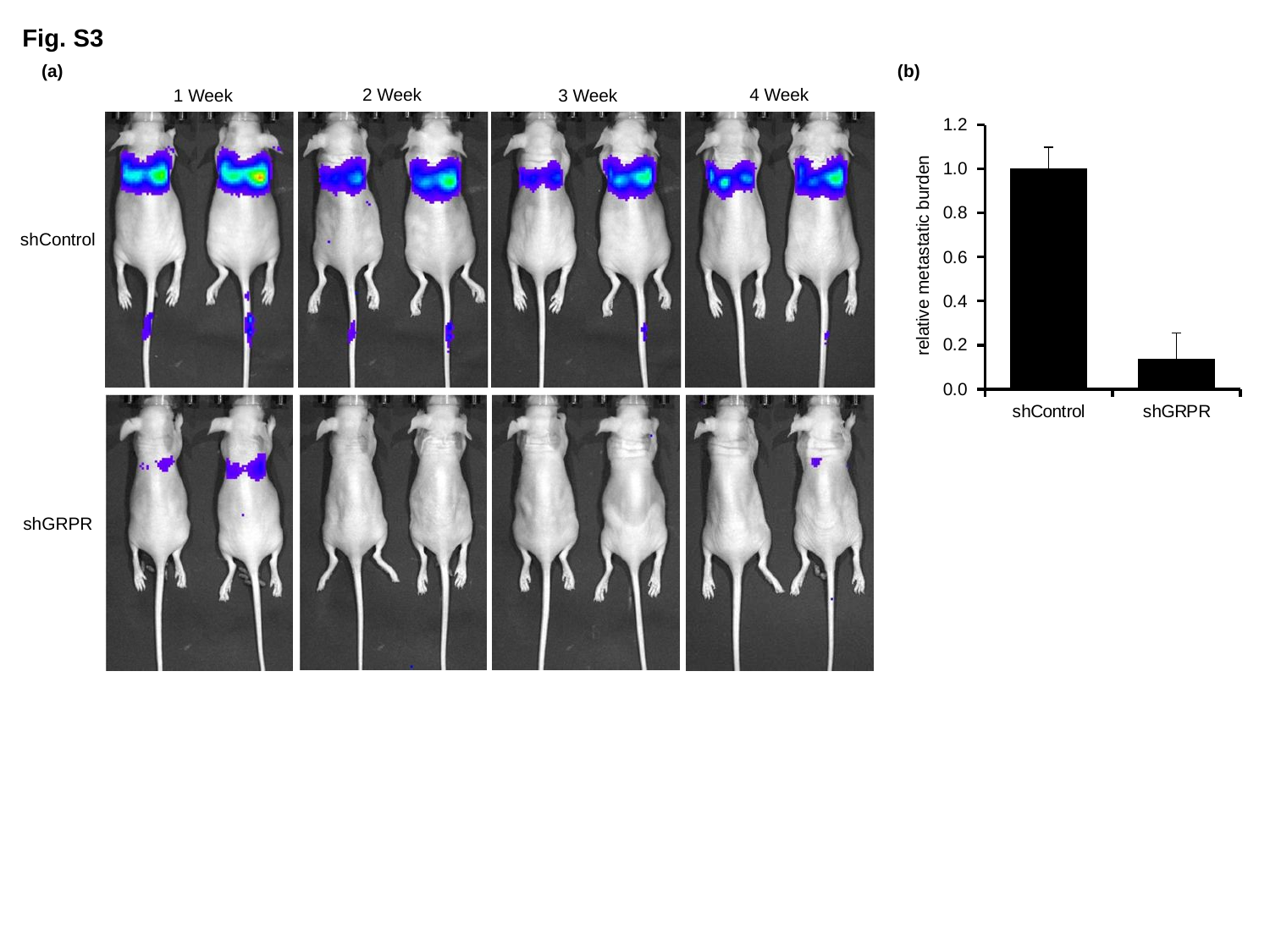

Fig. S3
(b)
(a)
2 Week
4 Week
1 Week
3 Week
### Chart
| Category | |
|---|---|
| shControl | 0.9999999999999999 |
| shGRPR | 0.13497671516036522 |
shControl
relative metastatic burden
shGRPR

## Slide 4
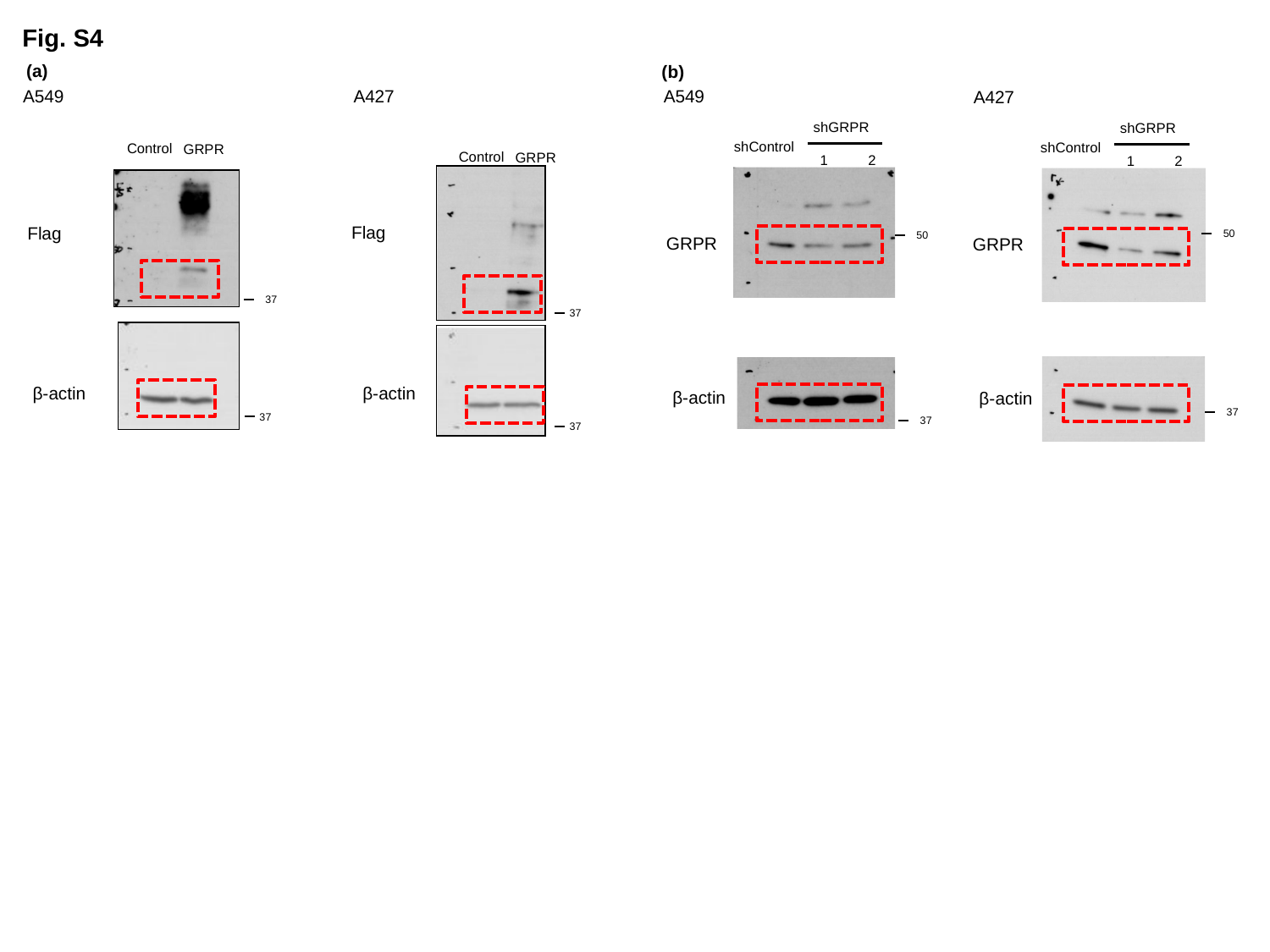

Fig. S4
(a)
(b)
A427
A549
A549
A427
shGRPR
shControl
GRPR
β-actin
 1 2
shGRPR
shControl
GRPR
β-actin
 1 2
Control
GRPR
Control
GRPR
Flag
Flag
50
50
37
37
β-actin
β-actin
37
37
37
37

## Slide 5
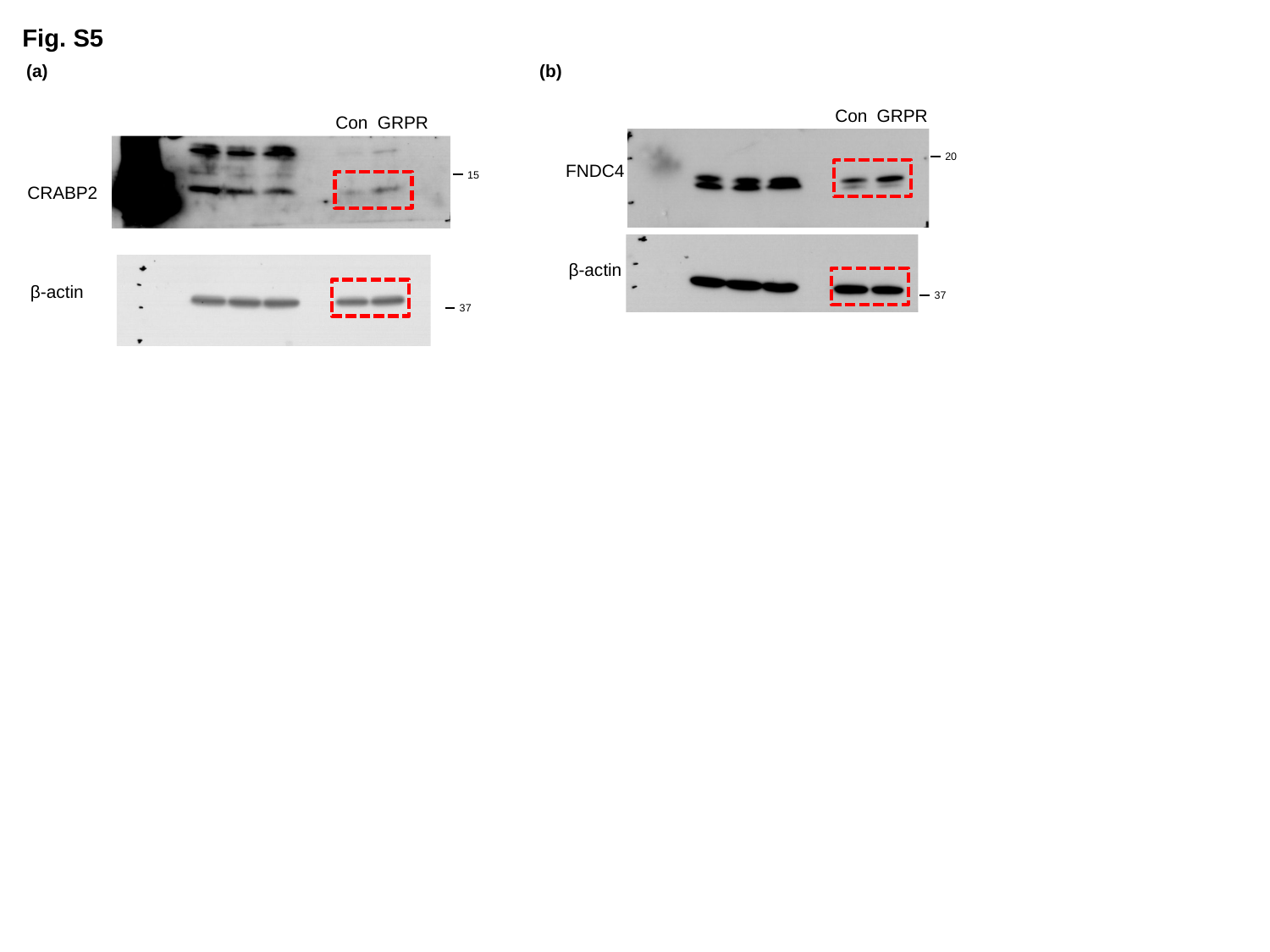

Fig. S5
(b)
(a)
Con GRPR
Con GRPR
20
FNDC4
15
CRABP2
β-actin
β-actin
37
37

## Slide 6
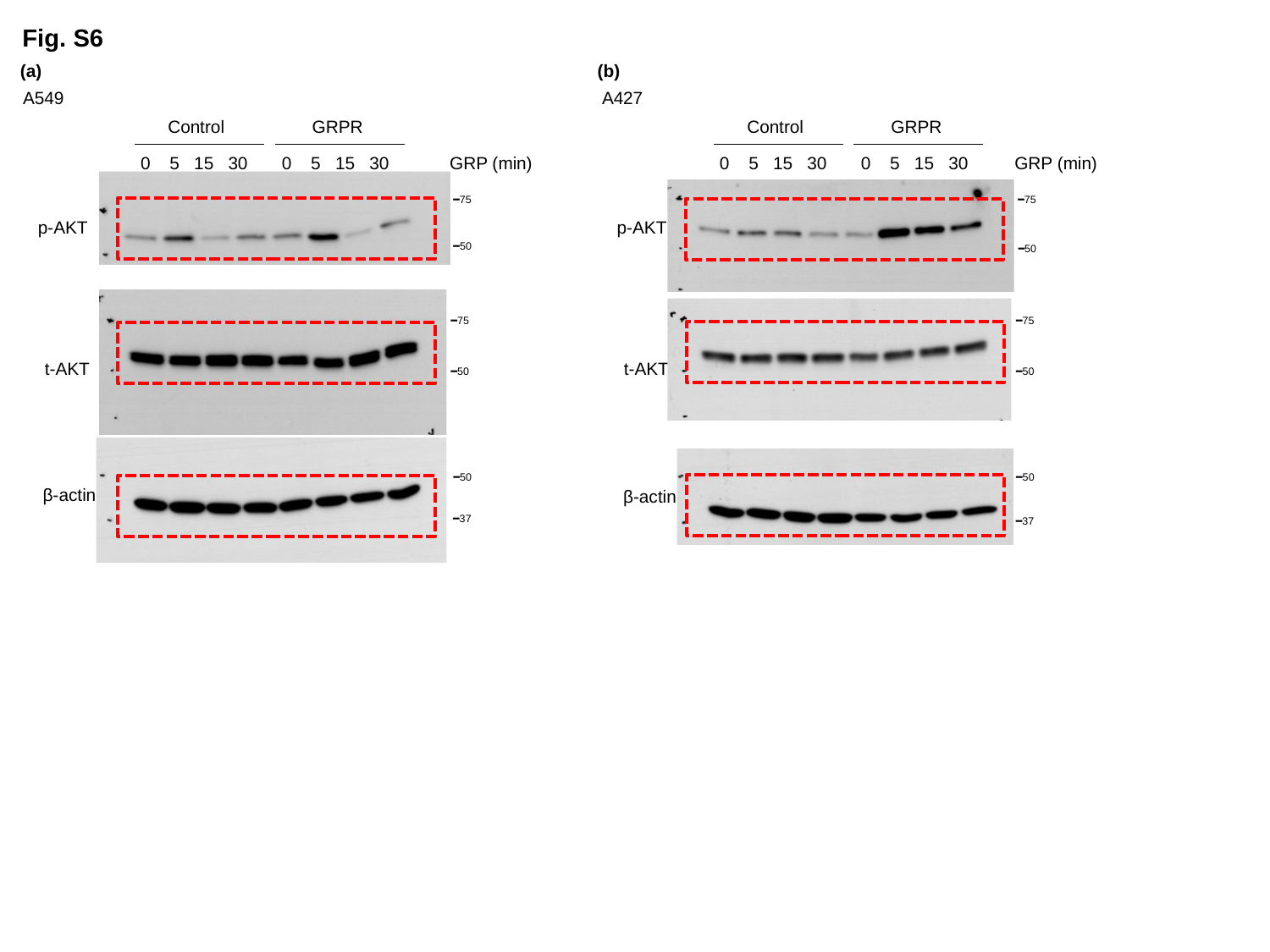

Fig. S6
(b)
(a)
A549
A427
Control
GRPR
Control
GRPR
0 5 15 30
0 5 15 30
GRP (min)
0 5 15 30
0 5 15 30
GRP (min)
━75
━75
p-AKT
p-AKT
 ━50
 ━50
━75
━75
t-AKT
t-AKT
━50
━50
━50
━50
β-actin
β-actin
━37
━37

## Slide 7
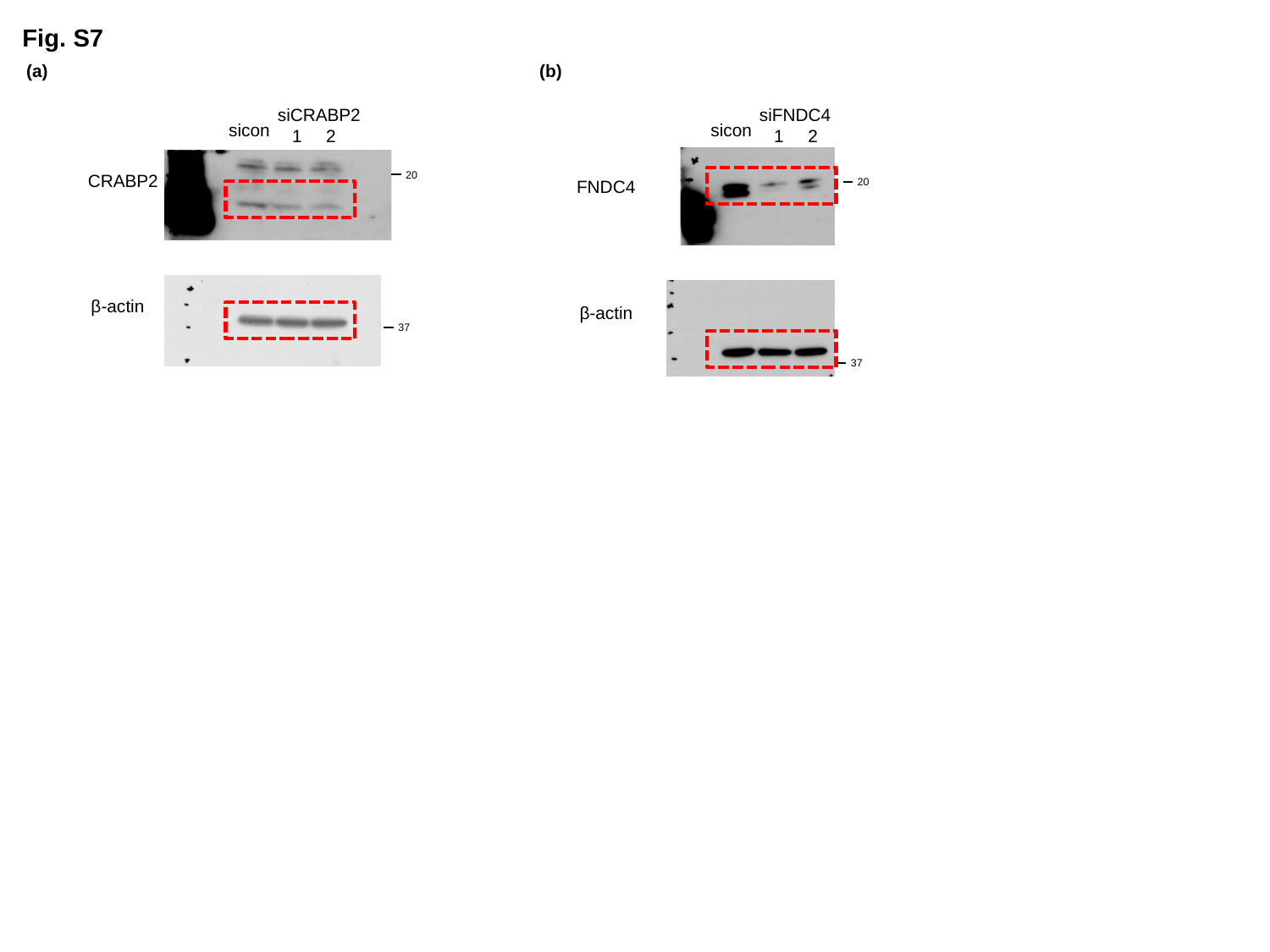

Fig. S7
(b)
(a)
siFNDC4
 1 2
siCRABP2
 1 2
sicon
sicon
20
CRABP2
20
FNDC4
β-actin
β-actin
37
37
